# Conference Report: METROLOGICAL ISSUES IN PRECISION-TOLERANCE MANUFACTURING: A REPORT OF A NIST IND USTRY-NEEDS WORKSHOP Gaithersburg, MD August 11–12, 1992

**DOI:** 10.6028/jres.098.020

**Published:** 1993

**Authors:** Dennis A. Swyt

**Affiliations:** Precision Engineering Division, National Institute of Standards and Technology, Gaithersburg, MD 20899-0001

## 1. The Goal of the Workshop

The purpose of this NIST-initiated workshop was to provide an opportunity for U.S. discrete-part manufacturing companies which see potential benefit from improved NIST research and services in areas of interest to them to make needs known such that NIST can address them, in particular to identify specific needed measurement-and-standards support from NIST which is not currently being provided or is being provided in forms or with accuracies insufficient to those needs.

## 2. The Motivation of the Workshop

When Congress changed the name of this Federal agency from NBS—the National Bureau of Standards—to NIST, it kept the NBS measurement-and standards function and added a new, principal mission: to assist U.S. industry to develop and deploy the new technology it deems it requires to achieve international competitiveness. Pursuing this mandate, the Director of NIST—who came to NBS from industry—is focusing on support which will assist U.S. manufacturers to achieve higher quality, lower costs, and increased speed to market. The Precision Engineering Division further focuses on providing to U.S. manufacturing industries practical access to national and international standards of length at levels of accuracy which they need.

The basis for the workshop—that is, its motivation, content, and structure—was the recent report by the Precision Engineering Division, entitled Challenges to NIST in Dimensional Metrology: The Impact of Tightening Tolerances in the U.S. Discrete-Part Manufacturing Industry, which identified a variety of industry needs within its mission area [1].

**Fig. 1:**
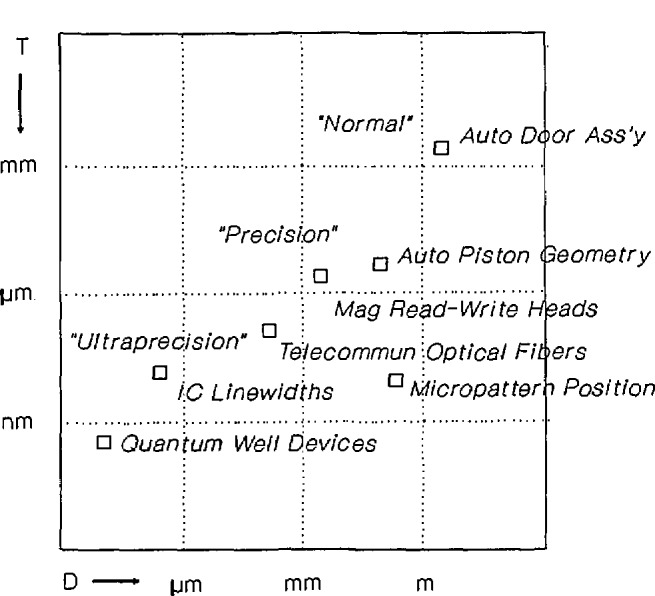
Log-log plot of tolerance versus dimension for discrete-part products in normal, precision, and ultraprecision regimes.

**Fig. 2:**
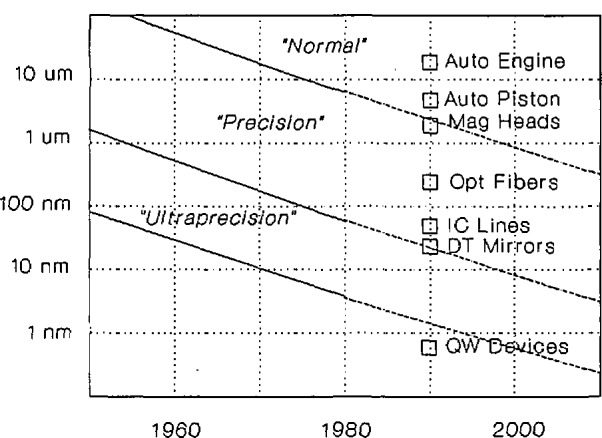
Semilog plot of trends in limiting values of tolerances in normal, precision and ultraprecision regimes with examples of state-of-art today.

**Fig. 3:**
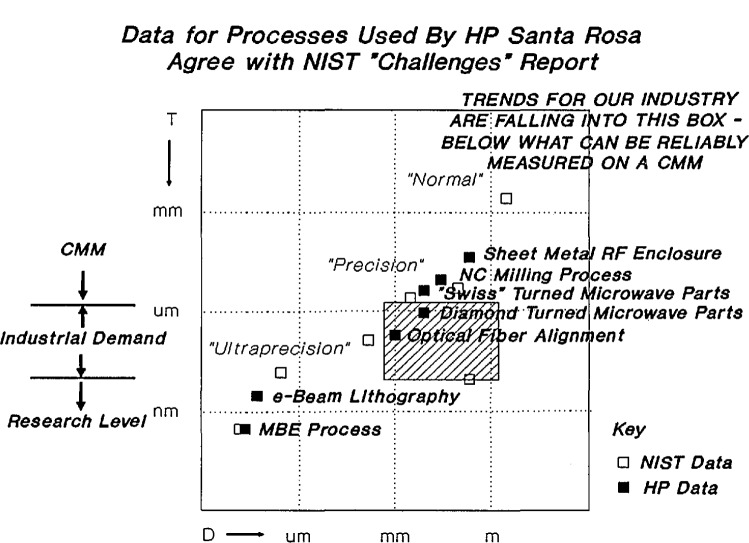
Graphical representation of industry measurement needs presented by working group 3.

This report showed an on-going trend to tighter dimensional tolerances in manufactured parts with: 1) the dimensions and tolerances of state-of-the-art precision-tolerance products shown in Fig. 1; 2) the decrease in the size of such tolerances an average of a factor of three every 10 years shown in Fig. 2; 3) the multiples by which accuracies of industry’s production control measurements are expected to be better than tolerances and the multiples by which accuracies of NIST measurements are expected by industry to be better than those of their production control shown in Table 1; and 4) the lag of NIST laboratory capabilities behind these changes in industry needs shown in Table 2.

## 3. The Scope of the Workshop

Manufacturing industries served by this workshop included those with products whose function, quality, cost and availability depend on parts or part features specified in terms of length-based tolerances.

Invited industry participants included those from firms in industries such as automotive, aerospace, farm-and-construction equipment, machine tool, electronics, semiconductor equipment, computers and instrumentation which have measurement problems associated with assuring that dimensional features of the product as made conform to those of the product as designed.

Attendees were company, industry, or agency representatives able to address specifics of measurement needs in terms of design tolerances, manufacturing deviations, production-control measurement accuracies, and required reference standards and in the context of their business or mission goals and needs. Among the 80 industry attendees were representatives of, for example, AT&T, Boeing, Caterpillar, Cummins Engine, Eastman Kodak, Ford, General Motors, Hewlett Packard, IBM, Ingersoll Milling, Northrup, Texas Instrument, the machine tool and gear manufacturers associations, and an array of instrument and gage manufacturers. A complete list of firms represented at the workshop is shown in Appendix A.

## 4. The Structure of the Workshop

The 2 day workshop included plenary and parallel working sessions organized to address the range of industries, measurement technologies and problems suggested by the “Challenges” report which defined the three tolerance regimes shown in Table 3.

Following welcoming remarks by Dr. John W. Lyons, Director of NIST, were three theme-setting presentations by representatives of Boeing commercial aircraft company (illustrating issues of close-tolerance manufacturing of large-scale discrete-parts in the aerospace industry), Cummins Engine company (illustrating issues of the close-tolerance manufacturing of medium-scale discrete-parts in the automotive industry), and IBM (illustrating issues of close-tolerance manufacturing of small-scale discrete-parts in the advanced microelectronics industry). Similar plenary-session presentations on the metrological issues in discrete-part manufacturing in their lines of business were made by representatives of 11 other firms.

During the course of the workshop, participants received briefings on NIST laboratory capabilities, the research and services it provides, and the new means by which it aims to support domestic manufacturing industries, including: Cooperative Research and Development Agreements (CRDAs), under which NIST can work jointly with industry on problems of mutual interest; Regional Manufacturing Technology Centers, the system of technology-transfer centers being funded and administered by NIST; and the Advanced Technology Program (ATP), the new grants program to help high-tech companies or consortia of companies improve their competitiveness. Attendees were also asked what effect on their operations NIST’s proposed adoption of the BIPM-recommended means of expressing measurement uncertainty would have (Appendix B).

Individual firm’s needs were presented to NIST in the form of worksheets. For developing common points of view on industry needs, attendees self-selected into one or more of five working groups topically devoted to:

1. Larger-Scale Parts, Coordinate Measuring Machines and Theodolites;
2. Complex-Shape Parts, Including Gears and Screw Threads;
3. Medium-Sized Parts, Including Small-Bore Features (such as microwave airlines);
4. Figure/Finish, Including Diamond Machining; and
5. Ultrasmail Features, Microelectronic Lithographies, and Scanning-Probe Microscopies.

## 5. Specific Working-Group Recommendations

The following is a summary of the industry needs and desired NIST actions related to those needs which the working groups produced and industry spokespersons presented in plenary sessions.

### 5.1 Working Group on Larger-Scale Features

This working group included a large contingent of users of coordinate measuring machines and their summary-report statement of needs consisted of: 1) improved NIST calibration accuracy, especially of meter-size standards, with a base calibration by NIST or DoE; 2) standards-committee support by NIST, especially ISO, where NIST should lead the U.S. delegation; 3) development of a long-term strategy to learn the accuracy achieved on real workpieces on 3D coordinate measuring systems; 4) for probing, provision of application documents on performance characterization and calibration methodology, covering also thermal properties and especially long-stylus properties; and 5) calibrations of thermal-expansion coefficients of CMM calibration artifacts to accuracies of one to three percent. For emphasis, this group concluded its presentation of needs by reading a letter from a Vice President of the Caterpillar Company to NIST, quoted in the “Challenges” report, saying:

“Five years ago our company embarked on a major factory modernization program with the purpose of maintaining, if not increasing the competitive edge that has historically been ours. A vital component of our overall program was modernization of our metrological equipment. Central to the metrology upgrade was increased use of coordinate measuring machines (CMMs) on our factory floor. Obtaining length standards of adequate accuracy, certified by NIST, for use in certification and ongoing verification of our new CMMs is a problem of ongoing concern. For the past three years we have been unable to obtain certification of step gages from NIST… Our current solution is to use PTB (German Standards Bureau) for certification of step gages … The current situation is unacceptable. We cannot afford the cost and time of continuing to send reference artifacts to Europe for certification. For both competitive and strategic reasons, we must have a metrologically strong partner at NIST…”

### 5.2 Working Group on Complex-Shape Parts

This working group included a large contingent of manufacturers of gears and gear-measuring equipment and their summary-report statement of needs consisted of: 1) establishing a calibration service for gear involute/lead masters for which none now exists, potentially including a round-robin measurement exercise coordinated by NIST; 2) publishing a report addressing the propagation of uncertainties down through the “hierarchies” of measurement/calibration processes; 3) doing something about the problem that “calibration” is a vague term with different meanings in different applications; 4) conducting round-robins, correlating data, and working with industry in developing measurement techniques because methods are needed to correlate measurement techniques for complex shapes; 5) with small manufacturers needing certification of outside measurement services, address the accreditation of organizations providing measurements of complex shape; and 6) step up research, including cooperative research with industry, in the area of on-line measurement techniques for processes producing parts of complex shape.

### 5.3 Working Group on Mid-Scale Features

This working group included representatives of a number of companies and groups within a single company who indicated measurement issues for part and feature sizes “below what can be reliably measured on a CMM,” a region which they showed graphically by amending Fig. 1, originally of the NIST “Challenges” report, to produce Fig. 3. The figure so presented illustrates three points: 1) state-of-the-art tolerances for manufacturing processes used by Hewlett-Packard Santa Rosa — from sheet-metal radio-frequency enclosures through diamond-turned microwave parts to e-beam and molecular-beam-epitaxy fabricated features — agree with those shown in the NIST “Challenges” report; 2) there is a region of industrial demand below that delivered by coordinate measuring machines and above that achieved in research; and 3) the trend of this industry’s needs lies in this region which is bounded by dimensions from sub-millimeter to above one meter and tolerances from about 20–50 nm to about 2 μm.

A summary of 18 specific types of artifact standards needed to support unique or non-general purpose measurement solutions for particular types and sizes of manufactured part features which was presented is shown as Table 4. In addition to those artifact standards, this working group also indicated industry need for educational/training information from NIST, including NIST-lead development of industry-useable calibration procedures, for precision-tolerance applications when production tolerances are less than 16 times NIST capabilities, i.e., measurement accuracies, including better documentation of NIST procedures.

### 5.4 Working Group on Figure/Finish

This working group included representatives of manufacturing companies with principal interests in characterization of surface roughness, surface figure (e.g., curvature of aspherical forms), and specialized production processes associated with them, such as single-point diamond machining and ductile regime grinding.

In the area of surface finish, the major concerns were that NIST should: 1) send NIST technical staff to participate in development of international (ISO) and national (ANSI) documentary standards dealing with surface roughness; 2) develop a set of artifacts and standard protocols for instrument evaluation, for example, sinsuoidals with spatial wavelengths from 200 mm on down and “realistic” (e.g., quasi-random) surfaces; 3) provide standard data sets for software evaluation; and develop better step-height standards. A specific surface-finish standard needed is one with standard data sets for known sinusoidal, triangular, and square-wave forms (for instrument adjustment) plus a random wave form with an RMS roughness of less than 10 nm (for instrument correlation).

In the area of figure, the present NIST program should be encouraged, with its good approach of progressing from successively developing state-of-the-art capabilities to measure first flat, then sphere, then asphere forms. In the area of ductile regime grinding, NIST needs to develop the means for measurement of sub-surface damage, including determination of the transfer function between instruments, and developing definitions and standard tests, all supported by educational services including tutorials and giving of hands-on experience.

### 5.5 Working Group on Ultrasmall-Scale Features

This working group included representatives of manufacturing firms that either use or produce scanning-probe-microscope instruments for the characterization of ultrasmall features of discrete-part manufactured goods such as nanoelectronic devices. The principal needs from NIST identified were those for: 1) Standard Reference Materials—with certification of sub-micrometer artifacts in all the dimensions (2D plus height) and 2D in distance (with field characterization and pattern placement); 2) Technology Development — especially STM probe tip development, which is a key enabling technology for small high-tech companies facing heavy foreign competition but having difficulty getting support from the NIST Advanced Technology Program; and 3) Road Maps—with a 5 year plan from NIST in response to the workshop’s requests, indicating what is feasible and how it will conform to other roadmaps in industry, e.g., Sematech.

## 6. Principal Findings of the Workshop

The principal recurring theme of needs, broadly shared among the industry representatives — whether from commercial aircraft, heavy equipment, automobiles, engines, computers, instrumentation or microelectronics was an urgent request that NIST provide new or improved accuracy physical artifact dimensional standards applicable to industry specific needs, including those for coordinate measuring machines, involute gears, small bore microwave devices, x ray optical surfaces and nanometer scale microelectronics.

The most frequently stated basis of the need of companies for more high-accuracy artifact standards from NIST is the “new traceability” required to meet ISO-9000-type quality requirements for products to be sold both in the European Economic Community and Pacific-rim nations. The next most frequently stated basis is the need for fixed reference points to support development of innovative products being strongly competed by Japan.

Finally, the sense of the closing plenary session was that this group of representatives of U.S. discrete-part manufacturing urged NIST to immediately respond to these stated needs for higher-accuracy artifact standards across the range of applications indicated in this workshop, including developing a documented operational plan spelling out how over the next few years it will do so.

## Figures and Tables

**Table 1:** Effect of two of the most commonly used ratios on accuracies of inspection and reference measurements: 1) the gage maker’s rule (factor of 10); and 2) the minimum ratio (factor of 4)

	Gage maker’s rule	Minimum ratio
Manufacturing tolerance	*T*	T
Inspection accuracy	*M* = *T*/10	*M* = *T*/4
Expected NIST accuracy	*N*=*M*/10 = *T*/100	*N* = *M*/4 = *T*/16

**Table 2:** NIST accuracy relative to that required to support design tolerances and production measurements at limit of normal-tolerance regime over the period of years 1970 to 2000

Measurement	Relations	1970s	1980s	1990s	2000
Manufacturing design tolerance	*T*	20 μm	7.5 μm	2.5 μm	1 μm
Production measurement accuracy	*M* = *T*/4	5 μm	1.75 μm	0.625 μm	0.25 μm
Rcq’d NIST measurement	*N*=*M*/4 emsp; = *T*/16	1.25 μm	0.5 μm	0.15 μm	0.05 μm
Realized NIST measurement accuracy	*U*	1.0 μm	Same		?

**Table 3:** Tightening of realizable dimensional tolerances in normal, precision, and ultraprecision tolerance regimes of machining from 1980 to 2000

Machining	Production and measuring	Accuracy	Accuracy
Normal	Conventional milling and turning coordinate measuring machines	7.5 μm	1 μm
Precision	Diamond turning machines interferometer systems	0.075 μm	0.01 μm
Ultraprecision	Atom and ion-beam machining scanning tunneling microscopes	0.005 μm	< 0.001 μm

**Table 4:** Artifact standards needed for unique or non-general purpose solutions

Dimensional feature type	Typical size	Improved standards needed
Outside diameter	1–1000 mm	CMM ball plates, step gages, gage blocks
Inside diameter	1–1000 mm	Master rings, step gages
Step height	0.1–100 μm	Amplification standards for step height
Distance between lines	0.001 mm–20 m	Laser interferometers, digital and line scales
Out-of-roundness	0.01–100 μm	Amplification standards for roundness
Layer thickness	0.01–100 μm	Foils test specimens
Angle, subdivision of circle	0–360 degrees	Levels, index tables
Angle — materialized	0–360 degrees	Right angles, polygons, angle blocks, cone angle
2D/3D dimensions	0.001–1000 mm	Two- and three-dimensional standards
Geometrical properties of involutes	Module 0.4–10 mm	Master wheels,
	Diameter 5–500 mm	Involute standards
Geometrical properties of screw threads	Diameter 1–200 mm	Thread masters, plugs, rings
Roughness, waviness	0.01–1000 μm	Parameter standards, comparison specimens
Straightness	0.01 μm	Straightness standards, straight edges
Flatness	0.01 μm	Flatness standards, surface plates
Roundness	0.01–100 μm	Roundness standards, CMM reference balls
Cylindrieity	0.01 μm	Plugs, rings
Shape of curves	0.01 μm	Curve standards
Shape of surfaces	0.1 μm	Surface standards

**Table 5:** Responses of workshop attendees on question of what effect would NIST changeover to CIPM system of representing measurement uncertainty would have on manufacturing firm^a^

Company	Effect^a^	Using	Comment
Automated Precision	o	3*σ* + se	
Boeing Aircraft	t	(2 or 3)*σ* + se	Some documentation problems
Cummins Engine	+	2*σ*	Meaningful use in uncertainty budgets
Cummins Engine	+	2*σ*	Align NIST with foreign national laboratories
Detroit Center Tool	o	3*σ* + se	Use precision in gage acceptance
Eastman Kodak	+	(2 or 3)*σ*	Criteria are cost, international political; need standard
Federal Products	o	3*σ* + se	In some cases it may be an advantage
GKS Inspection	o		Uses what customers want
GM Powertrain	+	3*σ*	
Ingersoll Mach Tool	+, t		Common method good
Lion Precision Gage		2*σ*	Not considered problem in depth
Moore Products		3*σ* + se	Not sure of customer response
Naval Aviation Depot	t	3*σ* + se	Should use only ISO Type A
Optra	o		Seems like good idea
Oak Ridge Natl Lab	o	3*σ* + se	Positive step
Precise Inspection	t	A = 2R	Any change important
Tencor Instruments	o	(1 or 3)*σ *	Used with appropriate reference
Timken	o	3*σ*	

a Key to symbols: + = The NIST changeover will provide an advantage to my firm. o = The NIST changeover will pose no problem. t = The NIST changeover will pose some transition problems. − = The NIST changeover will pose substantial problems.

## 7. Appendix A. Manufacturing Companies Represented at Workshop

AALA	Eastman Kodak
American Gear Mfrs	Edge Technologies
AT&T	EDM
AT&T Bell Labs	Federal Products
Automated Precision	Ford Motor Co.
Baxter Associates	General Motors
Boeing Aircraft	Giddings & Lewis
Boeing Defense	GKS Inspection
Brown & Sharpe	GM Powertrain
Caterpillar	Hewlett Packard
Cummins Engine	IBM
Detroit Center Tool	Industrial Tectonics
Diffracto	Ingersoll Milling
Digital Instruments	Machine
ITW Zero Systems	Precise Inspection
Leica	R.E. Smith
Lion Precision	SAMI
M&M Precision	Tencor
Martin Marietta	Texas Instruments
Moore Special Tool	Timken
Moore Products	TopoMetrix
NMTBA	Ultratech Stepper
Newport Klinger	Van Keuren
Northrup	VLSI Standards
Optodyne	Zygo
OPTRA	

## 8. Appendix B. Results of Representation of Uncertainties Survey

Also as part of the formal workshop, industry attendees were queried on the issue of NIST adoption of the CIPM recommendation on representation of uncertainties in measurement [2], specifically:
“*What is your view of NIST changing the basis of its ‘accuracy’ statements from the old system of ‘random-and-systematic added linearly’ to the new system of ‘type A and type B added sum-of-squares’, in particular from the predominantly used U_T_* = 3*σ* + *se to a proposed U_T_* = 2*σ*.”

Eighteen firms of the 75 which attehded the workshop responded. Results are summarized in Table 5 according to the legend shown with it.

In sum, 100% of the 18 respondees said it would pose no substantial problem (also, in effect, given the opportunity to do so none of the 75 attendees said it would pose a problem); 22% (4 respondees representing three firms and a DoD facility: Boeing Aircraft, Precise Inspection, Ingersoll Milling again and the Naval Aviation Depot at Pensacola) said the change would involve some transition problems. The only “transition problems” specified were “some problems with documentation” (Boeing). Five gaging companies, including Detroit Tool, Federal Products, Lion Gage, Moore Products, Precise Inspection, suggested that there is a special problem associated with how uncertainty of measuring gages is handled when assessing whether gaged parts are in or out of tolerances or when assessing whether the gage itself is in or out of tolerance.

Five respondees representing four firms (Cummins Engine, Eastman Kodak, General Motors, and Ingersoll Machine Tool) said the change to the CIPM recommended system of representing uncertainty would provide a positive advantage. Advantages noted were: “Financial and international political criteria” (Eastman Kodak); “Align NIST with other major foreign national laboratories” (Cummins); “Allow meaningful use of uncertainties in firm’s uncertainty budgets” (Cummins); “Provides common method to state/interpret ‘accuracy’ statements” (Ingersoll); and “Positive step toward internationally consistent measurements” (ORNL).
